# Pancreatic-specific autoantibodies to glycoprotein 2 mirror disease location and behaviour in younger patients with Crohn’s disease

**DOI:** 10.1186/1471-230X-12-102

**Published:** 2012-08-06

**Authors:** Dimitrios P Bogdanos, Dirk Roggenbuck, Dirk Reinhold, Thomas Wex, Polychronis Pavlidis, Ulrike von Arnim, Peter Malfertheiner, Alastair Forbes, Karsten Conrad, Martin W Laass

**Affiliations:** 1 Division of Transplantation Immunology and Mucosal Biology, King’s College London School of Medicine at King’s College Hospital, Denmark Hill Campus, Bessemer Road, London SE5 9RJ, UK; 2 GA Generic Assays GmbH, 15827, Dahlewitz, Berlin, L.-Erhard-Ring 3, Germany; 3 Institute of Molecular and Clinical Immunology, Otto-von-Guericke University Magdeburg, 39120 Magdeburg, Leipziger Str. 44, Germany; 4 Department of Gastroenterology, Hepatology and Infectious Diseases, Otto-von-Guericke University Magdeburg, 39120, Magdeburg, Leipziger Str. 44, Germany; 5 Department of Gastroenterology and Clinical Nutrition, University College Hospital, London, UK; 6 Institute of Immunology, Technical University Dresden, 01307, Dresden, Fetscherstraße 74, Germany; 7 Children’s Hospital, Technical University Dresden, 01307, Dresden, Fetscherstraße 74, Germany

**Keywords:** Autoantibody, Autoantigen, Autoimmunity, Crohn’s disease, Gastroenterology, Glycoprotein 2, Inflammatory bowel disease

## Abstract

**Background:**

Glycoprotein 2 (GP2) was discovered as the major autoantigen of Crohn’s disease (CD)-specific pancreatic autoantibodies (PAB). We investigated anti-GP2 IgA and IgG antibodies as novel serological parameters in CD and assessed their association with distinct disease phenotypes.

**Methods:**

Anti-GP2 and anti-Saccharomyces cerevisiae (ASCA) IgA and IgG were detected by ELISA employing recombinant human GP2 and phosphopeptidomannan, respectively and PAB by indirect immunofluorescence (IIF) in 271 sera, 169 with CD and 102 with ulcerative colitis (UC). As healthy controls 160 adult blood donors and 65 children were included.

**Results:**

Anti-GP2 IgG and/or IgA were more prevalent in CD (51/169, 30.2%) than in UC (9/102, 8.9%) patients and in controls (9/225, 4%) (p < 0.001 respectively). ASCA IgG and/or IgA were present in 60/169 (35.5%) in CD and in 7/102 (6.9%) in UC patients (p < 0.001). CD patients with ileocolonic location (L3) showed a significantly higher prevalence of anti-GP2 and ASCA IgA and/or IgG (40/113 and 48/113, respectively; p < 0.05 for both comparisons), whereas CD patients with colonic location (L2) revealed a significantly diminished prevalence for these autoantibody specificities (2/32 and 5/32, respectively, p < 0.05 for both). Anti-GP2 IgG were significantly more prevalent in CD patients with stricturing behaviour (B2) and perianal disease (7/11, p < 0.02) and less prevalent in those with penetrating behaviour (B3) and perianal disease (4/31, p < 0.05). The occurrence of anti-GP2 IgA and/or IgG was significantly more prevalent in CD patients with age at diagnosis of ≤16 years (16/31, p < 0.009). Prevalence of one or more anti-GP2 or ASCA IgA and/or IgG was significantly higher in L3, B2, and A1 and lower in L2 (68/113, 27/41, 23/31, 6/32; p < 0.04, respectively).

**Conclusions:**

Anti-GP2 IgG and IgA, constituting novel CD specific autoantibodies, appear to be associated with distinct disease phenotypes identifying patients at a younger age, with ileocolonic location, and stricturing behaviour with perianal disease.

## Background

The most prevalent clinical entities of inflammatory bowel disease (IBD), Crohn’s disease (CD) and ulcerative colitis (UC), affect as many as one in 250 individuals among Caucasians, and demonstrate an increase in other ethnic populations [1-4]. Although the pathophysiology of IBD is poorly understood, there is scientific evidence demonstrating that a damaged mucosal barrier is leading to mucosal inflammation triggered by intestinal bacteria in genetically predisposed individuals [3,5]. The intestinal inflammation in CD patients affects all layers of the bowel wall and adventitia and in contrast to UC is not confined to rectum and colon, but can be found throughout the alimentary tract [1].

Immune responses to disease-specific autoantigens also appear to be a prominent feature of CD, and are possibly involved in the pathogenesis of IBD [3]. Glycoprotein 2 (GP2) has recently been identified as the major autoantigenic target of CD-specific pancreatic autoantibodies (PAB) [6-8]. GP2 is mainly expressed as glycosyl phosphoinositol (GPI) membrane-anchored protein in the pancreas and released together with zymogens into the duodenum upon hormonal or neuronal stimulation [9,10]. Interestingly, GP2 has also been demonstrated to be a membrane-anchored receptor of microfold cells (M cells) in human intestinal Peyer’s patches (PP) and to be over-expressed at the site of CD inflammation in contrast to UC [6,11]. GP2 is involved in transcytosis of bacterial antigens, the presentation of whom by dendritic cells give rise to an antigen-specific immune response [11]. GP2 interacts with epithelial and activated T cells, binds to scavenger receptor on endothelial cells, and modulates innate and adaptive immune responses supporting a potential pathophysiological role [2,12].

Patients with IBD demonstrate disease-specific antibodies that may aid in the differential diagnosis of IBD, especially in the case of unclassified IBD [13,14]. Antibodies to bacterial peptides and glycans have been considered diagnostic markers of IBD but their prognostic significance is a matter of debate [15-17]. While the diagnostic significance of anti-GP2 antibodies in IBD has been studied in some detail, the clinical significance of these autoantibodies is unclear [2,18-21]. Thus, it is currently unknown whether humoral autoreactivity to GP2 or PAB respectively may assist in the prediction or stratification of disease activity or whether these autoantibodies are associated with specific clinical features [2,19,21-23].

The aim of the present study was to investigate the association of anti-GP2 antibodies with disease characteristics in CD in comparison with anti-Saccharomyces cerevisiae antibodies (ASCA), an established serological marker of CD. Here, we provide evidence for the first time that humoral autoreactivity to GP2 in CD appears to be associated with distinct clinical phenotypes.

## Methods

### Patient population

Serum samples from 169 patients with CD (median of age: 36 years) and 102 patients with UC were collected at the Children’s hospital of the Technical University Dresden, at the Department of Gastroenterology, Hepatology, and Infectious Diseases of the Otto-von-Guericke University Magdeburg, and at the Department of Gastroenterology and Clinical Nutrition, University College Hospital, London. All samples were taken at the time of consent and enrolment. Demographic and clinical characteristics of patients with CD are shown in Table 1. In total, 225 controls (median of age: 35 years, min 9 months, max 85 years) were recruited: 165 healthy adult blood donors and 65 children, who were admitted for eye surgery correcting their strabismus but were otherwise healthy. As control group for the comparison with patients with onset of disease below 17 years, the 65 children of the control group (median of age: 6 years, min 9 months, max 16 years) were selected.

**Table 1:** Demographic and clinical characteristics of patients with Crohn’s disease

	**CD (n = 169)**
Female, n (%)	102 (60.3)
Median age at study (max,min)	36 (8,87)
Age at diagnosis (years)	
below 17 years (A1), n (%)	31 (18.3)
between 17 and 40 years (A2), n (%)	19 (11.2)
above 40 years (A3), n (%)	119 (70.4)
Location	
ileal (L1), n (%)	24 (14.,2)
colonic (L2), n (%)	32 (18.9)
ileocolonic (L3), n (%)	113 (66.9)
upper disease, modifier (L4), n (%)	12 (7.1)
Behavior	
non-stricturing, non-penetrating (B1), n (%)	86 (50.9)
stricturing (B2), n (%)	41 (24.3)
penetrating (B3), n (%)	42 (24.8)
perianal disease modifier (p), n (%)	62 (36.7)
non-stricturing, non-penetrating (B1p), n (%)	20 (11.8)
stricturing (B2p), n (%)	11 (6.5)
penetrating (B3p), n (%)	31 (18.3)

Patients with CD were assigned a behavioural phenotype according to the Montreal classification [24]. Patients with unclassified IBD were excluded from the study. Correctness of diagnosis had been confirmed for all patients by 3 investigators (UA, AF, and MWL). Median age of patients with UC was 47 years (minimum 17, maximum 92) and 57 from 102 patients were female (55.9%). The diagnoses of CD and UC were based upon standard clinical, radiological, endoscopical and histological criteria [25,26].

The study was approved by the local ethics committees and conducted in accordance with the Helsinki declaration. Written informed consent was obtained from each individual. Aliquots of serum samples have been stored at −80 °C until use.

### Detection of antibodies to GP2 by ELISA

Anti-GP2 (IgA and IgG) autoantibodies were detected in sera of patients employing an ELISA from Generic Assays (Dahlewitz/Berlin, Germany), in accordance with the manufacturer’s instructions. This ELISA is based on recombinant human GP2 expressed in *Spodoptera frugiperda* 9 cells as solid-phase antigen [18]. For expression of GP2 by the baculovirus system, the plasmid pcDNA3.1+GP2-trunc-Thrombin-His is used which codes the amino acid sequence of GP2 isoform BAA88166 (pancreatic zymogen granule membrane associated protein GP2 alpha form) corresponding to the official isoform 2 (NP_001493) without 8 amino acids at the N-terminal end [27].

The anti-GP2 IgG ELISA displayed an intra-assay variability of 5.7% and an inter-assay variability of 5.0% for sera with elevated concentrations of 24.3 U/ml and 26.9 U/ml, respectively. The anti-GP2 IgA ELISA revealed an intra-assay variability of 5.9% and an inter-assay variability of 5.0% for sera with an elevated concentration of 17.8 U/ml and 18.5 U/ml, respectively. The functional assay sensitivities for anti-GP2 IgG and IgA determined as described elsewhere were assessed at 2.4 U/ml and 1.8 U/ml, respectively [28].

### Detection of antibodies to Saccharomyces cerevisiae by ELISA

In view of published studies reporting the frequent co-occurrence of CD-specific autoantibodies like PAB or in particular anti-GP2 and ASCA, and in order to compare anti-GP2 with ASCA, serum samples from patients were also tested for this anti-microbial reactivity. ASCA were determined as described previously, employing commercially available ELISA (ASCA IgA, ASCA IgG, GA Generic Assays GmbH, Dahlewitz, Germany) according to the recommendations of the manufacturer as reported elsewhere [13].

The intra-assay coefficient of variation was 2.0% for a sample containing 76 U/ml and 2.8% for a sample containing 78 U/ml of ASCA IgA and ASCA IgG, respectively. The inter-assay coefficient of variation was 5.9% for a sample containing 72 U/ml and 1.8% for a sample containing 81 U/ml of ASCA IgA and ASCA IgG, respectively.

### Assessment of pancreatic antibodies (PAB)

Antibodies to exocrine pancreas were detected by running patient samples on commercially available pancreas tissue sections according to the recommendations of the manufacturer (GA Generic Assays GmbH, Dahlewitz, Germany). Briefly, tissue sections were incubated in a moist chamber at RT for 30 minutes with 50 μl of serially diluted serum, starting at a dilution of 1 in 20 as reported elsewhere [18].

Samples were subsequently washed, embedded, and analysed with the automated interpretation system AKLIDES 40 (Medipan, Dahlewitz/Berlin, Germany) as described for other IIF tests [29]. Samples with a titre of 1:20 were considered borderline and samples with a titre of 1:40 or higher were scored positive.

### Statistical analysis

A Kolmogorov-Smirnov test was used to analyse the data for normality. Differences between groups were tested by Kruskal-Wallis and Fisher’s exact test with two-tailed probability. Spearman’s rank correlation test was applied for within group comparison. P values < 0.05 were considered significant. Assay performance including sensitivity, specificity, positive and negative likelihood ratio and receiver-operating characteristics (ROC) curve analysis were determined using Medcalc statistical software (Medcalc, Mariakerke, Belgium). The measured values were expressed as medians with 95% confidential intervals (CI).

## Results

### Patient cohorts

To investigate humoral autoreactivity to GP2 in IBD, 271 patients with IBD (169 CD patients, 102 UC patients) and 225 controls were included into this multicentre study with one British and two German gastroenterology departments. Patient characteristics are depicted in Table 1.

### Comparison of antibodies to GP2 with PAB in IBD patients and controls

Recombinant human GP2 immobilised on microtiter plates was employed to detect anti-GP2 IgG and IgA antibodies in sera of 169 patients with CD, 102 patients with UC and 225 controls (Table 2).

**Table 2:** Antibody distribution in 169 patients with Crohn’s disease (CD), 102 patients with ulcerative colitis (UC), and 225 controls

	**CD**		**UC**	**controls**
	**All CD patients (n = 169)**	**ASCA negative CD patients (n = 109)**	**(n = 102)**	**(n = 225)**
Anti-GP2 IgA, (%)	22 (13.0)	9 (8.2)	2 (2.0)	3 (1.3)
Anti-GP2 IgG, (%)	48 (28.4)	25 (22.9)	8 (7.8)	6 (2.7)
Anti-GP2 IgA and/or IgG, (%)	51 (30.2)	26 (23.9)	9 (8.8)	9 (4.0)
PAB > = 1/20, (%)	74 (43.8)	45 (41.3)	24 (23.5)	nd
PAB > = 1/40, (%)	65 (38.5)	38 (34.9)	21 (20.6)	nd
ASCA IgA, (%)	39 (23.1)	-	3 (2.9)	2 (0.9)
ASCA IgG, (%)	48 (28.4)	-	5 (4.9)	7 (3.1)
ASCA IgA and/or IgG, (%)	60 (35.5)	-	7 (6.9)	8 (3.5)
ASCA and/or anti-GP2, (%)	86 (50.9)	-	15 (14.7)	16 (7.1)
Number of positive antibodies detected by ELISA				
0	83 (49.1)	83 (76.1)	87 (85.3)	208 (92.4)
1	40 (23.7)	18 (16.5)	12 (11.8)	13 (5.8)
2	28 (16.6)	8 (7.3)	3 (2.9)	4 (1.8)
3	11 (6.5)	-	0 (0.0)	0 (0.0)
4	7 (4.1)	-	0 (0.0)	0 (0.0)

ASCA, antibody to mannan of *Saccharomyces cerevisiae*; CI, confidence interval; GP2, zymogen granule membrane glycoprotein 2; nd, not done; PAB, pancreatic autoantibody.

There was a significantly higher prevalence of anti-GP2 IgA (22/169, 13%), IgG (48/169, 28.4%) and IgA and/or IgG (51/169, 30.2%) in CD patients compared to UC patients (2/102, 2.0%; 8/102, 7.8%; 9/102, 8.8%, respectively) and in controls (3/225, 1.3%; 6/225, 2.7%; 9/225, 4%; respectively) in accordance to the Kruskal-Wallis test (p = 0.001, respectively).

Prevalence rates for anti-GP2 IgA [7/66 (10.6%) *vs* 15/103 (14.6%)] and IgG [16/66 (24.2%) *vs* 32/103 (31.1%)] as well ASCA IgA [16/66 (24.2%) *vs* 23/103 (22.3%)] and IgG [17/66 (25.8%) *vs* 31/103 (30.1%)] were not significantly different comparing German and English patients with CD, respectively. Pancreatic autoantibodies detected by IIF also demonstrated a higher prevalence in CD compared to UC patients [65/169 (38.5%) *vs* 21/102 (20.6%); p < 0.003] and were significantly correlated with anti-GP2 IgG (Spearman’s coefficients of rank correlation [rho] = 0.466, 95% interval of confidence [CI]: 0.367–0.554, p < 0.001). However, in UC patients the prevalence of PAB at titres ≥1:40 was significantly higher (21/102, 20.6%) compared to anti-GP2 IgG (8/102, 7.8%) and even with anti-GP2 IgG and/or IgA (9/102, 8.8%) (p < 0.02 and p < 0.03, respectively). In CD patients the difference in the occurrence of PAB ≥ 1:40 (65/169, 38.5%) and anti-GP2 (IgG: 48/169, 24.8% IgG and/or IgA: 51/169, 30.2%) did not reach significance (p = 0.064 and p = 0.108, respectively). There is good accordance between anti-GP2 and anti-PAB positivity. From 51 CD patients with anti-GP2 IgA and/or IgG positivity 45 (88%) showed also PAB positivity. Conversely, from 65 patients with PAB positivity 20 were negative for both anti-GP2 IgA and IgG respectively.

### Comparison of antibodies to GP2 with ASCA in IBD patients

**Figure 1:**
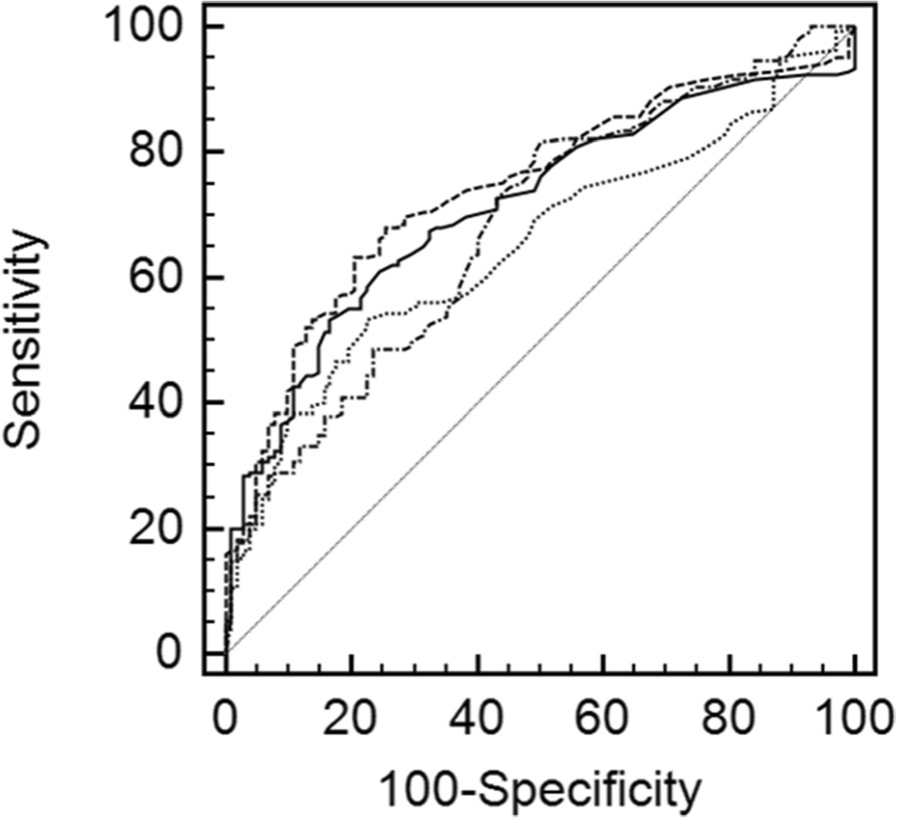
**Receiver-operating characteristic (ROC) curve analysis of anti-GP2 IgA.** Receiver-operating characteristic (ROC) curve analysis of anti-GP2 IgA and IgG as well as ASCA IgA and IgG detected in 169 CD patients and 102 UC patients: The area under the curve (AUC) was 0.652, 0.682, 0.713, and 0.743 for anti-GP2 IgA, anti-GP2 IgG, ASCA IgA, and IgG, respectively. The AUC values were significantly different for the comparison of anti-GP2 IgA and ASCA IgG (p = 0.0262). All other AUC values were not significantly different. dashed line - ASCA IgG. solid line - ASCA IgA. dashed dotted line - anti-GP2 IgG. dotted line - anti-GP2 IgA.

To compare anti-GP2 reactivity with an established antibody marker in IBD patients, ASCA IgA and IgG were determined in both patient cohorts. Like anti-GP2 antibodies, ASCA IgA (39/169, 23.1%), IgG (48/169, 28.4%), and IgA and/or IgG (60/169, 35.5%) demonstrated a significantly higher prevalence in CD than in UC patients [3/102 (2.9%), 5/102 (4.9%), 7/102 (6.9%), respectively; p < 0.001, for all comparisons] and in controls (Table 2). Receiver operating characteristics curve analysis revealed similar AUC values for the detection of ASCA and anti-GP2 isotypes (Figure 1). Only the comparison of anti-GP2 IgA and ASCA IgG showed significantly different areas under the curve (AUC) values (difference between areas: 0.0886, 95% CI: 0.0105–0.167; p < 0.03).

IgA and IgG reactivity to GP2 and Saccharomyces cerevisiae were significantly correlated (rho = 0.422, 95% CI: 0.340–0.533; rho = 0.335, 95% CI: 0.225 to 0.436, p < 0.001, respectively). However, 26 (23.9%) of ASCA negative patients demonstrated either IgA or IgG reactivity to GP2 (Table 2).

### Assay performance characteristics for anti-GP2, ASCA, and PAB

Assay performance characteristics for the detection of anti-GP2 IgA and IgG were compared to corresponding ASCA and PAB values and the results are summarized in Table 3. Regarding the serological discrimination of CD and UC, ASCA and anti-GP2 antibodies alone have medium positive likelihood ratios (LR) indicating the ratio between the probability of a positive test result given the presence of the disease (+LR) and the probability of a positive test result given the absence of the disease (-LR). Interestingly, PAB demonstrated an even lower +LR despite the higher prevalence in CD patients.

**Table 3:** Performance characteristics of anti-GP2 and ASCA IgA/IgG and PAB investigating 169 CD patients and 102 UC patients

	**sensitivity**	**95% CI**	**specificity**	**95% CI**	**+LR**	**95% CI**	**-LR**	**95% CI**
anti-GP2 IgA	13.0	8.3–19.0	98.0	93.0–99.8	6.6	1.6–27.4	0.9	0.8–1.0
anti-GP2 IgG	28.4	21.7–35.8	92.2	85.1–96.6	3.6	1.8–7.3	0.8	0.7–0.9
anti-GP2 IgA or IgG	30.2	23.4–37.7	91.2	83.9–95.9	3.4	1.8–6.6	0.8	0.7–0.9
ASCA IgA	23.1	17.0–30.2	97.1	91.6–99.4	7.8	2.5–24.7	0.8	0.7–0.9
ASCA IgG	28.4	21.7–35.8	95.1	88.9–98.4	5.8	2.4–14.1	0.8	0.7–0.8
ASCA IgA or IgG	35.5	28.3–43.2	93.1	86.4–97.2	5.2	2.5–10.9	0.7	0.6–0.8
PAB ≥1/20	45.4	37.6–53.4	76.5	67.0–84.3	1.9	1.3–2.8	0.7	0.6–0.8
PAB ≥1/40	38.5	31.1–46.2	79.4	70.3–86.8	1.9	1.2–2.9	0.8	0.7–0.9
at least 1 ab*	50.9	43.1–58.6	85.3	76.9–91.5	3.5	2.1–5.6	0.6	0.5–0.7
at least 2 ab*	27.2	20.7–34.6	97.1	91.6–99.4	9.2	3.0–29.0	0.8	0.7–0.8
at least 3 ab*	10.6	6.4–16.3	100.0	96.4–100.0	∞		0.9	0.8–0.9
at least 4 ab*	4.1	1.7–8.3	100.0	96.4–100.0	∞		1.0	0.1–1.0

Sensitivity, specificity and likelihood ratios were calculated using a cut-off of 20 U/ml for all ELISA and a titre of 1/20 and 1/40 of IIF.

ab, antibody; ASCA, antibody to mannan of *Saccharomyces cerevisiae*; CD, Crohn’s disease; CI, confidence interval; ELISA, enzyme-linked immunosorbent assay; GP2, zymogen granule membrane glycoprotein 2; LR, likelihood ratio; PAB, pancreatic autoantibody; UC, ulcerative colitis; *anti GP2 and/ or ASCA.

The combination of ASCA and anti-GP2 antibody assessment improves the +LR values regarding the serological differentiation of CD and UC. The detection of at least 2 ELISA-antibodies thereof reveals a +LR for CD of 9.2. Only 3/102 (2.9%) patients suffering from UC displayed antibody reactivities scoring positive in two of the four ELISAs. The determination of 3 or more antibodies seen in 18/169 CD patients is highly specific for CD (100%) resulting in an infinite +LR.

### Association of anti-GP2 and ASCA with location of disease

Employing the Montreal classification of IBD, CD patients were stratified according to age at diagnosis, location, and behaviour of disease (Table 1). Fisher’s exact test revealed a significantly higher prevalence of IgA and/or IgG reactivity to GP2 (40/113, 35.4%) in CD patients with ileocolonic location of disease (L3) (11/56, 19.6%; p < 0.05) (Table 4).

**Table 4:** **Association of anti-GP2, ASCA, and PAB with disease characteristics in CD:*p*values for elevated (bold) or diminished (italic) prevalence of samples with anti-GP2, ASCA, or PAB in 169 CD patients detected by ELISAs and IIF, respectively**

	**anti-GP2**				**ASCA**				**PAB**	
	**IgA**	**IgG**	**IgA or IgG**	**IgA and IgG**	**IgA**	**IgG**	**IgA or IgG**	**IgA and IgG**	**> = 1/20**	**> = 1/40**
L1										
L2	*<0.02*	*<0.002*	*<0.001*	*<0.03*			*<0.02*			*<0.05*
L3		**<0.05**	**<0.05**			**<0.05**	**<0.002**			
L4										
B1										
B2										
B3		<*0.02*	*<0.04*							
B1p										
B2p		**<0.02**	**<0.02**							
B3p		*<0.05*								
A1		**<0.004**	**<0.009**	**<0.001**		**<0.004**			**<0.002**	**<0.001**
A2										
A3										*<0.02*

In contrast, there was a significantly lower prevalence thereof in CD patients characterized by a colonic location of disease (L2) [2/32 (6.2%) *vs* 49/137 (35.8%); p < 0.001]. A similar pattern was seen for ASCA IgA and/or IgG [L3: 48/113 (42.5%) *vs* 12/56 (21.4%); L2: 5/32 (15.6%) *vs* 55/137 (40.1%); p < 0.001, p < 0.02; respectively). While a significantly lower prevalence in CD patients with L2 was also detected for anti-GP2 IgA [0/32 (0.0%) *vs* 22/132 (16.7%)], anti-GP2 IgG [2/32 (6.2%) *vs* 46/137 (33.5%)], and the simultaneous appearance of both isotypes [2/32 (6.2%) *vs* 49/137 (35.8%)], only the occurrence of anti-GP2 IgG was significantly higher in CD patients with L3 [38/113 (33.6%) *vs* 10/56 (17.9%)] (Table 4). In contrast, the increased prevalence of PAB in CD with L3 [49/113 (43.4%) vs 16/56 (28.6%)] did not reach significance and only the occurrence thereof in CD with L2 was significantly reduced [7/32 (21.9%) *vs* 58/137 (42.3%); p < 0.05].

### Association of anti-GP2 and ASCA with behaviour of disease

Crohn’s disease patients with penetrating disease (B3) demonstrated a significantly lower prevalence of anti-GP2 IgG alone and of anti-GP2 IgA and/or IgG [6/42 (14.3%) *vs* 42/127 (33.1%) and 7/42 (16.7%) *vs* 44/127 (34.6%); p < 0.02, p < 0.04, respectively] (Table 4). The diminished appearance of anti-GP2 IgA (4/42, 9.5%) did not reach significance. Interestingly, CD patients with stricturing and perianal disease (B2p) showed an elevated occurrence of anti-GP2 IgG alone and of anti-GP2 IgA and/or IgG [7/11 (63.6%) *vs* 41/158 (25.9%) and 7/11 (63.6%) *vs* 44/158 (27.8%); p < 0.02, respectively]. Interestingly, there was also a significant lower prevalence of anti-GP2 IgG in CD patients with penetrating and perianal disease [4/31 (12.9%) *vs* 44/138 (31.9%), p < 0.05]. In contrast, there was no significant association for ASCA and PAB with disease behaviour detectable.

Apart from a significantly higher prevalence of CD specific IgG in patients with B2p (9/11, 81.8%) compared to IgA (2/11, 18.2%; p < 0.03), there was no further difference in the isotype prevalence regarding anti-GP2 or ASCA.

### Association of anti-GP2 and ASCA with age at diagnosis

Crohn’s disease patients with an age less than 17 years at diagnosis (A1) demonstrated a significantly higher prevalence of CD-specific IgG antibodies [anti-GP2 IgG 16/31 (51.6%) *vs* 32/138 (23.2%), ASCA IgG 15/31 (48.4%) *vs* 33/138 (23.9%), PAB 22/31 (71.0%) *vs* 43/138 (31.2%); p < 0.004, p < 0.004, p < 0.001, respectively]. Interestingly, the combination of IgA and/or IgG to GP2 also revealed a significantly higher prevalence in CD patients with A1 [16/31 (51.6%) *vs* 35/138 (25.4%), p < 0.009]. Remarkably, CD specific IgG occurred less prevalent in CD patients with an age at diagnosis above 40 years [anti-GP2 IgG: 2/19 (10.5%) *vs* 46/150 (30.7%); ASCA IgG: 4/19 (21.1%) *vs* 44/150 (29.3%)], however, only the less prevalent appearance of PAB reached significance in this patient group [2/19 (10.5%) *vs* 63/150 (42.0%); p < 0.02]. Although anti-GP2 IgA was more prevalent in CD patients with A1 than ASCA IgA [10/31 (32.3) *vs* 9/31 (29.0%)] and less in CD patients with A3 (1/19, 5.3% *vs* 2/19, 10.5%), all prevalence rates were not significantly different compared with the respective patient groups regarding age at diagnosis. To exclude a possible bias regarding higher prevalence of antibody occurrence in younger individuals, we selected a group of controls with an age below 17 years. There were no significant different prevalences of CD specific antibodies compared to the 160 adult blood donors. However, the prevalence of CD specific antibodies was significantly higher in the CD patient group consisting of 31 individuals with A1 than in this particular control group [anti-GP2 IgA: 10/31 (32.3%) vs 1/65 (1.5%), anti-GP2 IgG 16/31 (51.6%) vs 2/65 (3.1%), ASCA IgA: 9/31 (29.0%) vs 1/65 (1.5%), ASCA IgG 15/31 (48.4%) vs 1/65 (1.5%); p < 0.001, respectively].

### Association of the combined analysis of anti-GP2 and ASCA with disease phenotypes

Positivity for antibodies to GP2 or Saccharomyces cerevisiae in at least one of the four ELISAs investigated was significantly associated with ileocolonic disease location [68/113 (60.2%) *vs* 18/56 (32.1%); p < 0.001], stricturing behaviour [27/41 (65.9%) *vs* 59/128 (46.1%); p < 0.04], and age at diagnosis below 17 years [23/31 (74.2%) *vs* 63/138 (45.7%), p < 0.006] in CD patients (Table 5). Occurrence of at least one positive antibody was significantly less prevalent in CD patients with colonic location [6/32 (18.8%) *vs* 80/137 (58.4%); p < 0.001]. Remarkably, the highly specific occurrence of three or more antibodies in CD (n = 18) was significantly associated with ileocolonic disease location [16/113 (14.2%) vs 2/56 (3.6%); p < 0.04] and young age (A1) in CD [7/31 (22.6%) *vs* 11/138 (8.0%); p < 0.03].

**Table 5:** **Association of number of anti-GP2 and/or ASCA antibodies in one sample with disease characteristics in CD:*p*values for elevated (bold) or diminished (italic) prevalence of samples with differing numbers of anti-GP2 and/or ASCA in 169 CD patients detected by ELISAs with a cut-off of 20 U/ml**

	**Number of antibodies detected by ASCA and anti-GP2 ELISA**			
	**at least one**	**at least two**	**at least three**	**Four**
L1				
L2	*<0.001*	*<0.05*	*<0.03*	
L3	**<0.001**		**<0.04**	
L4				
B1				
B2	**<0.04**			
B3				
B1p				
B2p				
B3p				
A1	**<0.006**	**<0.007**	**<0.03**	**<0.003**
A2				
A3				

## Discussion

The major findings of the present study are that anti-GP2 antibodies are present in approximately 30% of patients with CD, and appear to identify cases with an earlier onset, ileocolonic location, and stricturing behaviour with perianal disease. Our findings support the notion that anti-GP2 antibodies are diagnostically and clinically-relevant markers of CD and can assist physicians in the management of patients with clinical suspicion of IBD. Not only anti-GP2 antibody testing by ELISA shows a remarkable positive correlation with PAB by IIF but also appears to allow better discrimination of low titre or borderline positive PAB by IIF and seems therefore more specific for CD than PAB testing. This supports the notion that anti-GP2 antibody detection can be a supplementary tool for the testing of CD-specific pancreatic antibodies complementing or even replacing the laborious IIF technique [2].

Cohorts from clinical centres recruiting patients participating in the present study reported a ~30% anti-GP2 seropositivity in patients with CD [18-20]. However, accurate estimation of the overall prevalence of anti-GP2 antibodies in patients with IBD could not be carried out up so far, as testing of serum samples was performed in different laboratories and at various time points [18-20]. The systematic approach used in the present study, employing simultaneous testing of all coded samples in one laboratory and by the same immunodiagnostician (DRo), allowed for continuity of testing, thus permitting safer conclusions regarding the exact frequency of anti-GP2 antibodies in IBD. The approximately 30% anti-GP2 seropositivity rate may indeed be an underestimation of the real prevalence in newly diagnosed cases with CD, if antibodies are diminished over the course of the disease as an effect of the administration of biological agents and immunosuppressive treatment for CD [2,18,19]. In fact, sharp decline of IgG anti-GP2 antibodies has been described in CD during a 12-month course with infliximab. Intriguingly, such conversion from seropositivity to seronegativity was not accompanied by simultaneous reduction of ASCA [19].

An experienced group of Investigators from Belgium have tested 164 patients with CD for anti-GP2 antibodies, using the commercially-available assay which was used in the present study [21]. Op De Beéck et al. reported a sensitivity of anti-GP2 antibodies in patients with CD of 21%. However, it is not clear at what time points these serum samples were tested for autoantibody reactivity [21]. Also, the overall prevalence of PAB in the Belgian CD cohort was significantly lower than that of the present study (31% vs. 44%), and therefore a lower prevalence of anti-GP2 in the Belgian IBD population was not a surprising finding. The strikingly negative correlation between lower gastrointestinal tract localization of Crohn’s disease and presence of anti-GP2 was a characteristic feature of both studies. Finally, Op De Beéck et al. have failed to associate anti-GP2 seropositivity with other clinical correlates, when we have found that the presence of these antibodies is more prevalent in patients who present with CD at a younger age, in addition to those with stricturing disease behaviour. Immediate comparisons of the statistical values amongst the two studies cannot be made, as the Belgian study has been published in the form of a short Letter to the Editor with limited access to the wealth of the statistical analyses’ data [19]. Even if there was a consensus amongst the two studies, it would be very premature to comment on the clinical significance of anti-GP2 antibodies in IBD, as the clinical relevance of these antibodies cannot be assessed based on two studies. Care must be exercised also, when extrapolating conclusions regarding the effect of biological agents in the behaviour of anti-GP2 antibodies as the Belgian investigators did not find a profound effect of infliximab and adalimumab in patients followed up for 6–44 months [19,21].

Exchange of serum samples among researchers and large, multi-centre, prospective studies are needed to better delineate the diagnostic and clinical relevance of anti-GP2 pancreatic antibodies and their behaviour over the course of the disease in patients with inflammatory bowel diseases.

The development of a commercially-available ELISA based on recombinant human GP2 will allow for the accurate detection of GP2-specific pancreatic autoantibodies in routine laboratory practice and the initiation of longitudinal studies [18]. It would be of interest to know whether such studies will provide independent verification of the thesis supported by the present data that combined anti-GP2 and ASCA testing performs better than relying on tests limited to ASCA alone.

## Conclusions

Anti-GP2, constituting novel CD specific autoantibodies, is positive in about 30% of patients with CD and their detection may be of clinical significance. Anti-GP2 IgG and IgA, constituting novel CD specific autoantibodies, appear to be associated with distinct disease phenotypes identifying patients at a younger age, with ileocolonic location, and stricturing behaviour with perianal disease.

## Abbreviations

ASCA: Antibody to mannan of Saccharomyces cerevisiae; BD: Blood donor; CD: Crohn’s disease; CI: Confidence interval; CV: Coefficient of variation; ELISA: Enzyme-linked immunosorbent assay; FAS = Functional assay sensitivity; GPI: Glycosyl phosphoinositol; GP2: Zymogen granule membrane glycoprotein 2; IBD: Inflammatory bowel disease; IIF: Indirect immunofluorescence; LR: Likelihood ratio; M cell: Microfold or membranous cell; OD: Optical density; PAB: Pancreatic autoantibody; PP: Peyer’s patches; rho: Spearman’s rank coefficient of correlation; ROC: Receiver operating characteristics; RT: Room temperature; UC: Ulcerative colitis.

## Competing interests

Dirk Roggenbuck is a shareholder of GA Generic Assays GmbH and Medipan GmbH. The remaining authors declare that they have no competing financial interests.

## Authors’ contributions

Study concept and design, analysis and interpretation of data, drafting of the manuscript: Dimitrios P. Bogdanos; Study concept and design, acquisition of data, analysis and interpretation of data, drafting of the manuscript: Dirk Roggenbuck; Subject recruitment, acquisition and analysis of data: Dirk Reinhold and Thomas Wex; Subject recruitment, acquisition and analysis of data: Polychronis Pavlidis; Subject recruitment, interpretation of data: Ulrike von Arnim, Peter Malfertheiner; Subject recruitment, interpretation of data and manuscript preparation: Alastair Forbes; Study concept and design, acquisition of data, analysis and interpretation of data, drafting of the manuscript: Karsten Conrad; Subject recruitment, interpretation of data and manuscript preparation: Martin W. Laass. All authors read and approved the final manuscript.

## Authors’ information

Alastair Forbes, Karsten Conrad and Martin W Laass shared last authorship.

## Pre-publication history

The pre-publication history for this paper can be accessed here:

http://www.biomedcentral.com/1471-230X/12/102/prepub
